# Vitamin D toxicity in a pediatric toxicological referral center; a cross-sectional study from Iran

**DOI:** 10.1186/s12887-020-02240-4

**Published:** 2020-07-20

**Authors:** Fariba Farnaghi, Hossein Hassanian-Moghaddam, Nasim Zamani, Narges Gholami, Latif Gachkar, Maryam Hosseini Yazdi

**Affiliations:** 1 Departments of pediatrics, Loghman Hakim Hospital, School of Medicine, Shahid Beheshti University of Medical Sciences, South Kargar St, Makhsos St,, Tehran, Iran; 2 Department of Clinical Toxicology, School of Medicine, Shahid Beheshti University of Medical Sciences, Tehran, Iran; 3 Social Determinants of Health Research Center, Shahid Beheshti University of Medical Sciences, Tehran, Iran; 4 Infectious and Tropical Diseases Research Center, Shahid Beheshti University of Medical Sciences, Tehran, Iran; 5 Kish Institute of Science and Technology, Tehran, Iran

**Keywords:** Vitamin D, Toxicity, Pediatrics

## Abstract

**Background:**

Vitamin D is an essential element for body health with its supplements generally administered to prevent vitamin D deficiency. Since these supplements are available in domestic settings, vitamin D toxicity may happen in children.

**Methods:**

All children younger than 12 years who presented to the pediatric emergency department of Loghman Hakim Hospital, Tehran, Iran with history of ingestion of more than 1500 IU/day of vitamin D supplements were enrolled. Patients’ demographic data, on-presentation signs and symptoms, laboratory findings, treatments given, and outcome were evaluated.

**Result:**

Fifteen patients presented during the study period. Their mean age was 46.53 ± 10.14 months and 12 (80%) were girls. All of them had unintentionally ingested vitamin D. Mean ingested dose was 406700.7 ± 227400.1 IU. In eight patients (53.3%), 25 hydroxy vitamin D level was more than 100 ng/mL. One patient experienced hypercalcemia while all of them were asymptomatic and discharged without complications. There was no significant difference between patients with and without high levels of 25 OH vitamin D regarding lab tests, toxicity course, and outcome.

**Conclusions:**

It seems that acute vitamin D toxicity is a benign condition in our pediatric population which may be due to high prevalence of vitamin D deficiency in Iran.

## Background

Vitamin D is a fat-soluble pro-hormone primarily synthesized in skin by sun-light exposure [1, 2]. Dairy products, fish, and mushrooms may also contain small amounts of vitamin D [3, 4].This vitamin has a crucial role in the health of the musculoskeletal system. Furthermore, vitamin D has beneficial effects on cardiovascular, respiratory, and immune systems [4–12].

Due to issues including heavy clothing, air pollution, reduced exposure to direct sunlight, inadequate nutrition, and lack of access to vitamin D-rich food especially among children, vitamin D deficiency is a common health problem in our country [13–15]. Thus, consumption of vitamin D containing supplements is encouraged in Iran during the recent years making them more available for unintentional poisonings in children although the frequency of poisoning with these supplements is still low compared to other poisonings in them [16–20]. Vitamin D toxicity may also happen as a result of inappropriate dose administration by physicians or errors in manufacturing or unlicensed vitamin D preparations [21–25].

Daily recommended dose of vitamin D supplements is reported to be 400 IU in infants, 600 IU in people younger than 70 years of age, and 800 IU in people over 70 [26–29]. Since studies in this regard are lacking in children, we aimed to assess all children with vitamin D toxicity referring to a tertiary referral center of toxicology during a year.

## Methods

In a retrospective descriptive cross-sectional study performed in Loghman Hakim Hospital between March 21st, 2018 and March 20th, 2019, all children younger than 12 years who presented to the pediatric emergency department with the history of vitamin D supplements ingestionof more than 1500 IU in a single occasion were enrolled in this study. Written informed consents were taken from the patients’ parents before case enrolment. After taking history and physical examination, blood tests were performed six hours post admission.

All demographic data (age, sex, weight, amount of vitamin D ingested), vital signs(blood pressure, heart rate, dehydration signs), symptoms on presentation (nausea, vomiting, abdominal pain, loss of appetite, irritation, headache, constipation, polyuria, polydipsia, fever, and growth retardation), laboratory findings (25 OH vitamin D, serum calcium, phosphate, alkaline phosphatase, urea, creatinine, urine Ca/Cr), treatments given (close observation, hydration, steroid, bisphosphonate), and outcome (recovery, death) were recorded and analyzed using statistical package for social sciences (SPSS) version 18.

This study was approved by the ethics committee of Shahid Beheshti University of Medical Sciences (IR.SBMU.RETECH.REC.1397.1216).

## Results

Fifteen patients presented during the study period. Their mean age was 46.53 ± 10.14 months (range; 24 to 60 months). Twelve patients (80%) were girls (male/female ratio was 1:4).Patients were referred 2.5 ± 1.6 h (range; 0.5 to 5 h) after the vitamin D supplement ingestion. had all ingested vitamin D supplements unintentionally and accidentally (oral 50000-IU vitamin D pearls). Mean ingested dose was 8.13 ± 4.54 pearls (range; 3 to 18 pearls) or 406700.7 ± 227400.1 IU (range;150000 to 900000 IU). One patient had serum calcium level of 12.5 mg/dL. She had ingested500000 IU vitamin D. After six hours of hydration, her serum calcium was normal. Patients’ information is mentioned in Table 1.

**Table 1:** Vitamin D dose and biochemical features in individual patients

Cases	Vit D dose (✕50000 IU)	Ca (mg/dl)	25-OH vit D(ng/ml)
1	10	12.5	10
2	3	10.5	35.5
3	4	9.5	48.3
4	5	8.7	55
5	8	10.2	56
6	4	10.5	65
7	8	10.5	97.3
8	17	8.9	102
9	3	10.4	104
10	6	9.7	107
11	10	10.4	111
12	7	10.1	120
13	9	10.2	120
14	18	9.8	138
15	10	9.3	500

Eight (53.3%) cases had 25 OH vitamin D levels more than 100 ng/mL. Mean serum 25-OH vitamin D was 111.3 ± 113.6 (range; 10 ng/mL to 500 ng/mL). There was no significant difference between variables in patient with and without high level of 25-OH vitamin D. The most important data is mentioned in Tables 2 and 3.

**Table 2:** Lab parameter features in patients (Mean ± SD)

Group		25-OH vit D (ng/ml)	Ca (mg/dl)	P (mg/dl)	ALK-P (U/L)	Cr (mg/dl)	Urea (mg/dl)	Ca/Cr Urine random
25-OH vit D < 100 ng/ml	Mean *N* = 7	52.4	10.3	4.8	478.0	0.4	26.7	0.2
	Std. Deviation	26.7	1.2	0.7	31.1	0.3	10.8	0.2
25-OH vit D > 100 ng/ml	Mean *N* = 8	162.9	9.8	4.7	546.0	0.6	23.7	0.1
	Std. Deviation	136.7	0.5	0.6	38.5	0.1	6.1	0.2
Total	Mean *N* = 15	111.3	10.1	4.7	526.6	0.6	24.6	0.2
	Std. Deviation	113.6	0.9	0.6	47.4	0.2	7.3	0.2

**Table 3:** Demographic features in patients (Mean ± SD)

Group		AGE (month)	Vit D dose (✕50000 IU)	BP systolic (CmH2O)	HR Beat/min	Weight (Kg)
25-OH vit D < 100 ng/ml	Mean *N* = 7	46.3	6.0	97.8	109.7	16.5
	Std. Deviation	12.8	2.6	6.4	5.1	3.4
25-OH vit D > 100 ng/ml	Mean *N* = 8	46.7	10.0	96.2	99.4	15.8
	Std. Deviation	8.0	5.2	7.9	8.6	0.6
Total	Mean *N* = 15	46.5	8.1	97.0	104.2	16.1
	Std. Deviation	10.1	4.5	7.0	8.8	2.3

None of them had signs and symptoms of vitamin D intoxication; 46.7% were observed for eight hours and received activated charcoal. Additionally, 53.3% were hospitalized and treated by activated charcoal and fluid therapy. All cases were discharged without any complications. All patients were taking vitamin D regularly. We recommended them to discontinue consumption of vitamin D supplements, keep low-calcium and vitamin D diet, take more liquid for at least one month, and recheck 25-OH vitamin D levels. Unfortunately, most of the patients did not refer for follow-up checkups.

## Discussion

A definite amount of vitamin D ingestion to cause toxicity has not been elucidated. Although maximum tolerable ingested dose is various in different age groups, maximum tolerable and safe dose of vitamin D is 1000 IU/day in infants younger than 6 months, 1500 IU/day in children older than 6 months, and 10000 IU/day in adults [27, 28, 30–33].

Vitamin D concentration is measured by 25- hydroxy vitamin D level because it has longer half-life compared to 1,25 OH vitamin D [27, 34]. Although there are different ranges of 25- OH vitamin D levels in several studies, the optimal level of 25-OH vitamin D is 30–100 ng/mL. Based on the normal range provided by the kit manufacturer, 25-OH vitamin D less than 10 ng/mL is deficient, while levels 11–30 ng/ml, 31–100, and over 100 ng/mL are insufficient, sufficient, and vitamin D toxicity, respectively [35].The most important laboratory findings in vitamin D toxicity are high levels of 25-OH vitamin D and hypercalcemia [34].

Patients with vitamin D toxicity may be asymptomatic or have signs or symptoms including nausea, vomiting, dehydration, abdominal pain, loss of appetite, irritation, headache, constipation, polyuria, polydipsia, fever, and growth retardation. In fact, most of the patients’ symptoms are compatible with high serum calcium level [32, 36]. Differences between symptoms and laboratory parameters in patients may be related to gastrointestinal absorption, vitamin D binding protein, vitamin D receptor (VDR), vitamin D storage, and diet [21, 32, 36]. All of our patients were asymptomatic and it might be due to the maximum tolerable intake dose differences in various groups and high prevalence of vit D deficiency among Iranian children [14, 32]. Also further studies are needed to be performed in this regard.

Vitamin D deficiency is estimated to be approximately 35% in boys and 65% in girls in Iranian pediatric population. Frequency of vitamin D insufficiency is reported to be 31%. It shows that vitamin D deficiency is a critical health problem among Iranian children. Recently, the prevalence of vitamin D toxicity has increased because of more availability of its over-the-counter supplements [37, 38].

This study is one of the first ones about vitamin D toxicity in Iranian children. Although there are various studies in this regard worldwide, most of them have been performed on adults. It describes all cases of pediatric vitamin D toxicity that had referred to a major center for treatment of poisoned children in Tehran, Iran within a year.

All of our patients were between 24 and 60 months of age and had unintentionally ingested 50000-IU vitamin D pearls. In fact, D-pearls are small, soft gelatin capsules with 50000 IU of vitamin D3 in each capsule that may be attractive for children. Also, accidental poisoning in this age is more common. In the majority of studies, poisoning happens more frequently in boys, but in this study, poisoning was more common among girls [39, 40]. This cannot be generalized to the whole population because our study is only a case series in one center during a year.

We observed all patients for eight hours in emergency room and received activated charcoal. Laboratory tests were checked six hours after vit D consumption. If they had hypercalcemia or 25-OH vit D more than 100 ng/ml, they were hospitalized and treated by activated charcoal and fluid therapy. Their serum calcium was rechecked after four hours of rehydration.

Some studies have demonstrated higher frequencies of symptoms and hypercalcemia in their patients compared to ours reporting hypervitaminosis D in adults and a few children, whereas in this study more than half of our cases (53.3%) had high levels of 25-OH vitamin D and just one had hypercalcemia without any symptoms who was hydrated. It may be due to high prevalence of vitamin D deficiency in Iranian children. On the other hand, vitamin D has long half-life because of its lipid solubility which leads to tendency for prolonged hypercalcemia. The absence of symptoms in our patients may be due to early laboratory parameters assessment mandating re-checking of the lab tests in consecutive hours post admission [19, 41–47].

One patient with hypercalcemia had 25-OH vitamin D level of 10 ng/ml; this might be due to the prozone or hook effect which is a known drawback in vitamin D measurement by Enzyme-linked Immunosorbent Assay (ELISA) method leading to falsely low vit D levels [48, 49].

We found only one study that had reported a patient with vitamin D intoxication in Iranian children. Faraht reported a 50-day-old girl in Imam Reza Hospital, Mashhad, Iran, with respiratory distress and hypotonia who had plasma creatinine of 1.4 mg/dL, BUN of 11 mg/dL, serum calcium level of 18.3 mg/dL, and 25-OH vitamin D of 75 ng/mL which were higher than normal range. It was due to the constant use of premature formula with high dose of vitamin D drop supplement (800 units /day) [50].

We had no access to the patients’ previous vitamin D levels and did not know if they were previously vitamin D-deficient or not. This is probably the most important limitation of the current study. Although there is not any reliable data on vitamin D toxicity among Iranian children, Spiller et al. have reported there were 25397 human exposures to vitamin D in USA in 2000–2014. It explains that between 2005 and 2011, vitamin D toxicity increased 1600%. There was 0.02% morbidity without mortality [51]. These results indicate that we need further researches in this field and vitamin D toxicity and vitamin D supplement intake should be more cautiously evaluated in children [45, 52–54].

## Conclusion

Although vitamin D toxicity is a rare poisoning among children, it is a critical condition due to serum calcium disturbances. It seems that acute vitamin D toxicityis a benign condition and has a good prognosis in Iranian pediatric population maybe due to high prevalence of vitamin D deficiency in Iranian children. Due to insufficient and limited studies among children, we need more studies in this regard.

## Data Availability

The datasets used and analysed during the current study are available from the corresponding author on reasonable request.

## Abbreviations

Vit D: Vitamin D
25OH vitamin D: 25-Hydroxy vitamin D
IU: International Unit
BUN: Blood urea nitrogen
mg/dl: Milligrams per deciliter
ng/ml: Nanograms per milliliter
ALK-P: Alkaline phosphatase
Ca: Calcium
P: Phosphor
HR: Heart Rate
BP: Blood Pressure
ELISA: Enzyme-linked Immunosorbent Assay
